# Narcissism predicts impulsive buying: phenotypic and genetic evidence

**DOI:** 10.3389/fpsyg.2015.00881

**Published:** 2015-07-07

**Authors:** Huajian Cai, Yuanyuan Shi, Xiang Fang, Yu L. L. Luo

**Affiliations:** ^1^Key Laboratory of Behavioral Science, Institute of Psychology, Chinese Academy of SciencesBeijing, China; ^2^Spears School of Business, Oklahoma State University, TulsaOK, USA

**Keywords:** impulsive buying, narcissism, maladaptive narcissism, adaptive narcissism, behavior genetics, twin study

## Abstract

Impulsive buying makes billions of dollars for retail businesses every year, particularly in an era of thriving e-commerce. Narcissism, characterized by impulsivity and materialism, may serve as a potential antecedent to impulsive buying. To test this hypothesis, two studies examined the relationship between narcissism and impulsive buying. In Study 1, we surveyed an online sample and found that while adaptive narcissism was not correlated with impulsive buying, maladaptive narcissism was significantly predictive of the impulsive buying tendency. By investigating 304 twin pairs, Study 2 showed that global narcissism and its two components, adaptive and maladaptive narcissism, as well as the impulsive buying tendency were heritable. The study found, moreover, that the connections between global narcissism and impulsive buying, and between maladaptive narcissism and impulsive buying were genetically based. These findings not only establish a link between narcissism and impulsive buying but also help to identify the origins of the link. The present studies deepen our understanding of narcissism, impulsive buying, and their interrelationship.

## Introduction

On November 11, 2014, the so-called “double 11” date, consumers from 217 countries spent 57.1 billion Chinese Yuan (about US $9.3 billion) on Alibaba, the largest Chinese online shopping site^1^, standing in sharp contrast to the site’s daily average purchase total. Why did so many consumers purchase so many items at this particular moment? Were the purchases planned in advance? We speculated that many people might have bought on impulse. Indeed, it is well documented that impulsive buying constitutes a large portion of daily purchases, particularly in this time of e-commerce (Kacen and Lee, 2002; Zhang and Shrum, 2009). In the U.S., for example, impulsive buying contributes US $4.2 billion to annual sales (Mogelonsky, 1998) and accounts for 50% of all mall purchases (Nichols et al., 2001). In the past half century, extensive research has been done on impulsive buying and established that impulsive buying could be either a spontaneous behavior triggered by situational factors, or a relatively stable tendency that varies across individuals (Rook, 1987; Rook and Fisher, 1995). In this research, we treated impulsive buying as a trait-like individual difference variable, and for the first time, investigated the relationship between narcissism and impulsive buying. We examined not only the phenotypic relationship between them, but also the genetic basis underlying this relationship.

### Impulsive Buying

Impulsive buying refers to a spontaneous and compelling purchasing behavior, which is usually characterized by lack of reflection and deliberation on one hand and immediate satisfaction and pleasure on the other hand (Rook, 1987; Kacen and Lee, 2002). Impulsive consumption can be neutral or even positive (e.g., a spontaneous gift for a sick friend). Since it violates rationality principles of human economics and most people have limited financial recourses, impulsive buying, actually, is often associated with negative outcomes, such as financial problems and post-purchase dissatisfaction and regret (Rook and Hoch, 1985; Rook, 1987). Given its prevalence and potential influences, impulsive buying has received much attention from researchers in various areas, such as in behavioral economics (e.g., Underhill, 1999), marketing (e.g., Applebaum, 1951), and psychology (e.g., Rook, 1987).

Early research on this subject mainly focused on external factors, such as product features (Applebaum, 1951) and shopping environments (Stern, 1962) that may facilitate impulsive buying. Later, researchers realized that personal dispositional factors of consumers could play a more important role in impulsive buying (Rook and Hoch, 1985; Rook, 1987), just as Rook and Hoch (1985, p. 23) have articulated: “It is the individuals, not the products, who experience the impulse to consume.” Most recently, researchers have identified many distinctive characteristics associated with impulsive buyers. Overall, they are more likely to be: (1) young rather than old and women rather than men (Dittmar et al., 1996; Wood and Ahuvia, 1998; Silvera et al., 2008); (2) high in hedonism, materialism, and individualism (Kacen and Lee, 2002; Zhang and Shrum, 2009); (3) high in extraversion, neuroticism, and impulsivity, but low in conscientiousness (Verplanken and Herabadi, 2001; Bratko et al., 2013; Lucas and Koff, 2014); and (4) low in self-control and self-regulation (Baumeister, 2002; Vohs and Faber, 2007).

Extant research suggests that although impulsive buying may be triggered by various situational factors, it is also clearly associated with fundamental individual differences, including age, values, personality, and the ability to inhibit impulses. Researchers have already established the trait nature of the tendency for impulsive buying and developed corresponding measures (Rook and Fisher, 1995; Verplanken and Herabadi, 2001). Moreover, a recent twin study showed that the tendency for impulsive buying is heritable (Bratko et al., 2013). Following this individual difference perspective, we examined the relationship between the tendency for impulsive buying and narcissism as well as its possible genetic bases.

### Narcissism and Impulsive Buying

Narcissism^2^ is typically characterized by a grandiose self-view. Narcissists^3^ are individuals who consider themselves to be special, entitled, superior to others, and at the same time, try to make others believe in this aggrandized self-image. Therefore, validating and promoting the inflated self constitutes an endless desire for narcissists. Habitually, they are ready to use all means to protect and elevate their excessively positive self (Morf and Rhodewalt, 2001). Among the various ways in which narcissists self-enhance, making purchases is an important one. Material possessions can promote a person’s apparent status, sustain a grandiose self-image, and influence others’ opinions of the individual (Sedikides et al., 2007; Cisek et al., 2008). Display of wealth can be an effective means to breed a favorable impression (Christopher and Schlenker, 2000). Overwhelmingly, narcissistic people exhibit high materialism (Rose, 2007) and strong aspirations for wealth, fame, and a positive image (Kasser and Ryan, 1996; Roberts and Robins, 2000), and tend to enhance their self-image by purchasing products, especially symbolic, exclusive, and personalized ones (Cisek et al., 2008; Lee et al., 2013).

In this article, we argue that narcissists are not only more likely to purchase, but also more likely to purchase on impulse. There are good reasons for us to hypothesize this tendency. First, narcissism is characterized by impulsivity (Raskin and Terry, 1988), the primary drive behind impulsive buying (Rook, 1987). A meta-analysis of 23 correlations between impulsivity and narcissism generated a mean effect size of *r* = 0.41 (Vazire and Funder, 2006; but see Miller et al., 2009). Second, impulsive buyers share several characteristics in common with narcissists, such as pursuing a positive self-identity (Dittmar et al., 1995, 1996; Dittmar and Drury, 2000), materialism, and individualism (Kacen and Lee, 2002; Zhang and Shrum, 2009). Third, narcissistic people are inclined to engage in compulsive buying, a pathological form of consumer behavior resembling impulsive buying in high materialism and low impulse control (O’Guinn and Faber, 1989; Rose, 2007; Ridgway et al., 2008). Consistent with these factors, empirical studies found that people with highly positive self-views liked to engage in impulsive buying to enhance their self-image (Cash and Cash, 1982) and people who exaggerated their attractiveness exhibited stronger impulsive buying tendencies (Lucas and Koff, 2014). To test the hypothesis that narcissism may predict the tendency for impulsive buying, we examined the relationship of impulsive buying with overall narcissism first and thereafter with its two components: adaptive narcissism and maladaptive narcissism.

### Adaptive Narcissism vs. Maladaptive Narcissism

In most studies among normal individuals, narcissism has been assessed by the Narcissistic Personality Inventory (NPI), which includes seven subscales of authority, entitlement, exhibitionism, exploitation, self-sufficiency, superiority, and vanity (Raskin and Terry, 1988). Yet in most cases, narcissism has been limited to being treated as a whole and has assumed to be maladaptive (Emmons, 1987; Hepper et al., 2013). In recent years, however, increasing evidence suggests that some components of narcissism, such as authority/leadership and self-sufficiency, are adaptive but some other components, such as exploitativeness, entitlement, and exhibitionism, are maladaptive (Watson and Biderman, 1993; Ackerman et al., 2011).

Thus far, research has established distinctions between adaptive and maladaptive narcissism. High adaptive narcissism has been shown to be associated with high conscientiousness, subjective well-being, self-esteem, assertiveness, self-confidence, and self-control, whereas high maladaptive narcissism is associated with high neuroticism, anxiety, social anxiety, depression, social maladjustment, Machiavellianism, impulsive antisociality, delinquency, aggression and low empathy, conscientiousness, and subjective well-being (Emmons, 1984; Raskin and Terry, 1988; Watson and Biderman, 1993; Washburn et al., 2004; Barry et al., 2007; Ackerman et al., 2011; Hepper et al., 2014). Based on these existing findings, we can see that it is maladaptive narcissism rather than adaptive narcissism that is associated with various negative outcomes. Therefore, we not only examined the relationship between impulsive buying and overall narcissism, but also the relationship between its two components, adaptive and maladaptive narcissism, and impulsive buying. We expected that maladaptive narcissism, instead of adaptive narcissism, would be predictive of impulsive buying.

### Heritability of Narcissism and Impulsive Buying

Behavioral genetic research has established that individual differences in narcissism are partly determined by genetic factors in both eastern and western cultures (Vernon et al., 2008; Luo et al., 2014). In addition, the connections between narcissism and personality traits, such as extraversion, openness, and conscientiousness, are largely due to common genetic influences (Vernon et al., 2008). Regarding impulsive buying, only one twin study has been done to date, its results showing that both impulsive buying and its associations with impulsivity, neuroticism, and extraversion are heritable (Bratko et al., 2013). No twin study, however, has simultaneously examined narcissism and impulsive buying, which constitutes a primary impetus for our work.

### Overview

In summary, we aimed to examine the relationship between narcissism and impulsive buying. We conducted two studies. While Study 1 capitalized on an online sample, Study 2 used a twin sample, which allowed us to investigate the genetic foundation of possible relationships between narcissism and impulsive buying.

## Study 1

### Method

#### Participants

One-hundred and twelve individuals (all Chinese, 47% male) recruited through a Chinese witkey website (i.e., www.zhubajie.com) participated in the online study. Their ages ranged from 17 to 38 years (*M* = 25.06, *SD* = 3.88). Every participant received CNY5 (about US $0.82) in compensation. The Ethics Committee of the Institute of Psychology, Chinese Academy of Sciences provided approval for the study. Additionally, we obtained written informed consent from all participants prior to commencing the test.

#### Measures

*Narcissism* was measured with the Narcissistic Personality Inventory (NPI; Raskin and Terry, 1988). The 40-item NPI has been successfully used in Chinese samples (Cai et al., 2012). Each item includes a pair of statements, one narcissistic and the other non-narcissistic as in the example, “I prefer to blend in with the crowd” (non-narcissistic statement) vs. “I like to be the center of attention” (narcissistic statement). For each item, participants indicated whether the narcissistic or non-narcissistic statement better described them. We coded the narcissistic statement choice as 1 and the non-narcissistic statement choice as 0. The internal consistency for the whole scale was desirable (α = 0.82). We calculated scores for adaptive and maladaptive narcissism based on previous decomposition of the NPI (Hepper et al., 2014). Specifically, 14 items reflecting authority and self-sufficiency were totaled to assess adaptive narcissism (α = 0.67), and 18 items concerning entitlement, exploitativeness, and exhibitionism were added up to index maladaptive narcissism (α = 0.61). The moderate internal consistencies of the subscales are congruent with past research, and in any case, have shown good construct validity and test–retest reliability (Barry et al., 2007; Barry and Malkin, 2010; Hepper et al., 2014).

*Impulsive buying* was measured with the Impulsive Buying Scale (Rook and Fisher, 1995). The scale includes nine items, such as the statement, “I often buy things spontaneously.” The participant indicated his or her agreement with these statements on a 7-point Likert scale (1 = *completely disagree*, 7 = *completely agree*). The scale was translated into Chinese and back-translation was used to ensure language equivalence. The scale was internally consistent (α = 0.86), and a mean score was calculated for each participant.

### Results

For the overall NPI score (*M* = 12.03, *SD* = 6.20), its relationship with the tendency for impulsive buying (*M* = 3.64, *SD* = 1.2) was positive but not significant (*r* = 0.14, *p* = 0.157). For the two subcomponents of narcissism, while the maladaptive narcissism score (*M* = 5.64, *SD* = 2.95) was positively correlated with impulsive buying (*r* = 0.20, *p* = 0.034), the adaptive narcissism was not (*M* = 4.15, *SD* = 2.63; *r* = 0.03, *p* = 0.784). In summary, although we failed to find a significant relation between overall narcissism and impulsive buying, we did found that maladaptive narcissism was significantly predictive.

## Study 2

### Method

#### Participants

One-hundred and fifty-two monozygotic (MZ) and one-hundred and fifty-two dizygotic (DZ; 94 same-sex, 58 opposite-sex) twin pairs sampled from the Beijing Twin Study (BeTwiSt) participated in the study. Twins in the BeTwiSt are socio-demographically representative of adolescents from Beijing, China (Chen et al., 2013). The ages of the twins in our sample ranged from 15 to 27 years (*M* = 18.29, *SD* = 1.96; 56% female). For 95% of the twin-pairs, we used DNA testing to determine zygosity, with classification accuracy approaching 100%; for the remaining 5%, we established zygosity by combining parent-reports and children’s self-reports on co-twin physical similarity and frequency of confusion, which reached a 90.6% accuracy rating (Chen et al., 2013). Thirty-five twin pairs did not complete the measure for impulsive buying. The data for one sibling of a DZ twin pair was missing and thus excluded in all analyses. The Ethics Committee of the Institute of Psychology, Chinese Academy of Sciences, provided approval for the study. Additionally, we obtained written informed consent from all participants and their parents prior to commencing the test.

#### Measures

The measures for narcissism and impulsive buying were the same as those in Study 1. The internal consistency for the entire NPI, adaptive narcissism, maladaptive narcissism, and impulsive buying, were all acceptable (α = 0.81, 0.63, 0.66, 0.89, respectively).

#### Genetic Analysis

We can estimate the additive genetic (A), shared environmental (C), and non-shared environmental (E) contributions to variance within a trait and covariance between traits by comparing the resemblance of MZ and DZ twin pairs for observed trait(s). MZ twins are 100% genetically identical, whereas DZ twins are 50% identical on average for additive genetic effects. The proportion of the variance of a trait, or the covariance between traits, explained by additive genetic effect, is referred as “heritability.” A shared environment increases the similarity of twins raised in the same family. A non-shared environment is unique to each individual, which also includes measurement error.

To estimate genetic and environmental effects, we employed univariate, bivariate, and multivariate models implemented in the OpenMx library (Boker et al., 2012) within the R statistical computing environment (R Development Core Team, 2012). First, the univariate model decomposed the variances of maladaptive narcissism and impulsive buying, respectively, into genetic (A) and environmental (C, E) effects. We examined the full ACE model first. Sub-models nested under the full model were also tested by systematically removing one component of variance.

**FIGURE 1:**
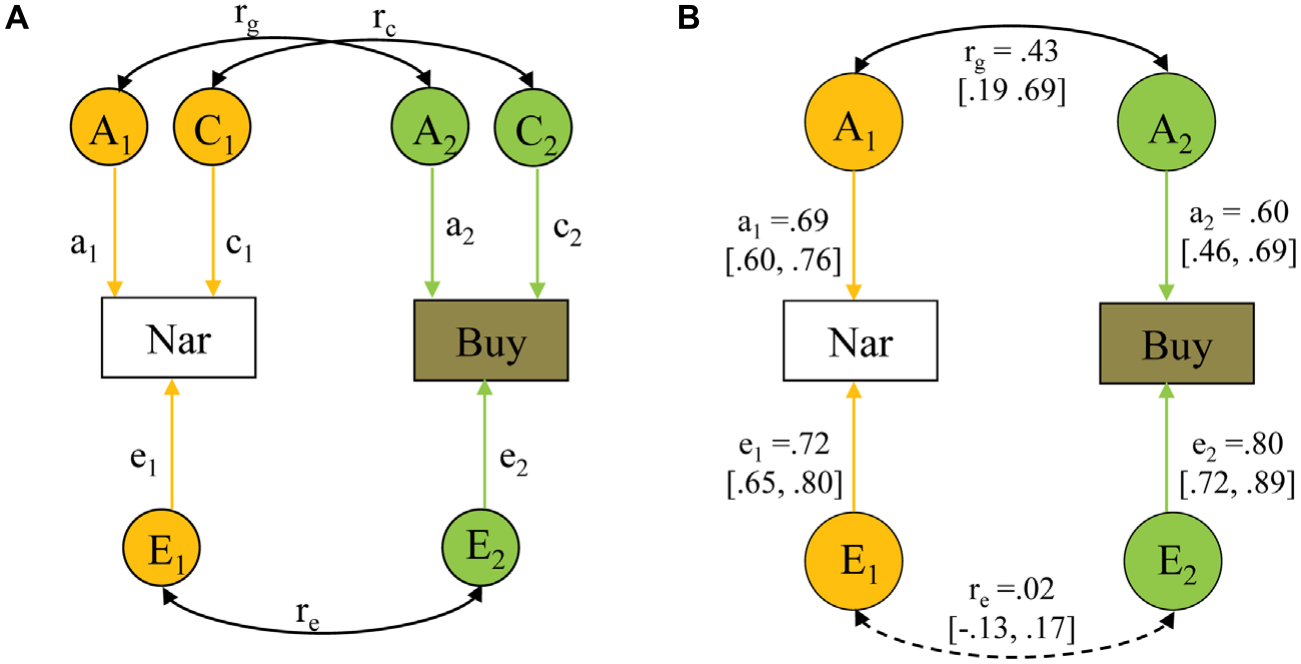
**Bivariate model-fitting for narcissism (Nar) and impulsive buying (Buy). (A)** Path diagram illustrating the bivariate model-fitting. **(B)** The best-fitting bivariate model. Measured variables are depicted in rectangles. Latent factors A (additive genetic factor), C (shared environmental factor), and E (non-shared environmental factor) are presented in circles. *r*_g_, genetic correlation; *r*_c_, shared environmental correlation; *r*_e_, non-shared environmental correlation. All path estimates (95% confidence intervals) are standardized but unsquared. The non-significant path is represented by a dashed line.

For the bivariate analysis of overall narcissism and impulsive buying, we used a correlated factors model based on Cholesky decomposition (Loehlin, 1996). Cholesky decomposition is similar to hierarchical regression analyses in non-genetic studies, through which the independent contribution of predictors entered later is assessed after controlling for the predictors entered earlier (Loehlin, 1996). Bivariate analysis allows the covariance of two traits to be partitioned into covariance that is due to additive genetic factors, common environmental factors and unique environmental factors. In this model, each variable is separately decomposed into its genetic, shared and non-shared environmental components at the same time that the correlations of these across variables are estimated (**Figure 1A**). A genetic correlation (*r*_g_) indicates the extent to which genetic influences on one trait overlap with those on the second trait (regardless of their individual heritabilities), just as in shared (*r*_c_), and non-shared (*r*_e_) environmental correlations. Similar to the univariate analysis, the full ACE model and the sub-models were systematically tested.

**FIGURE 2:**
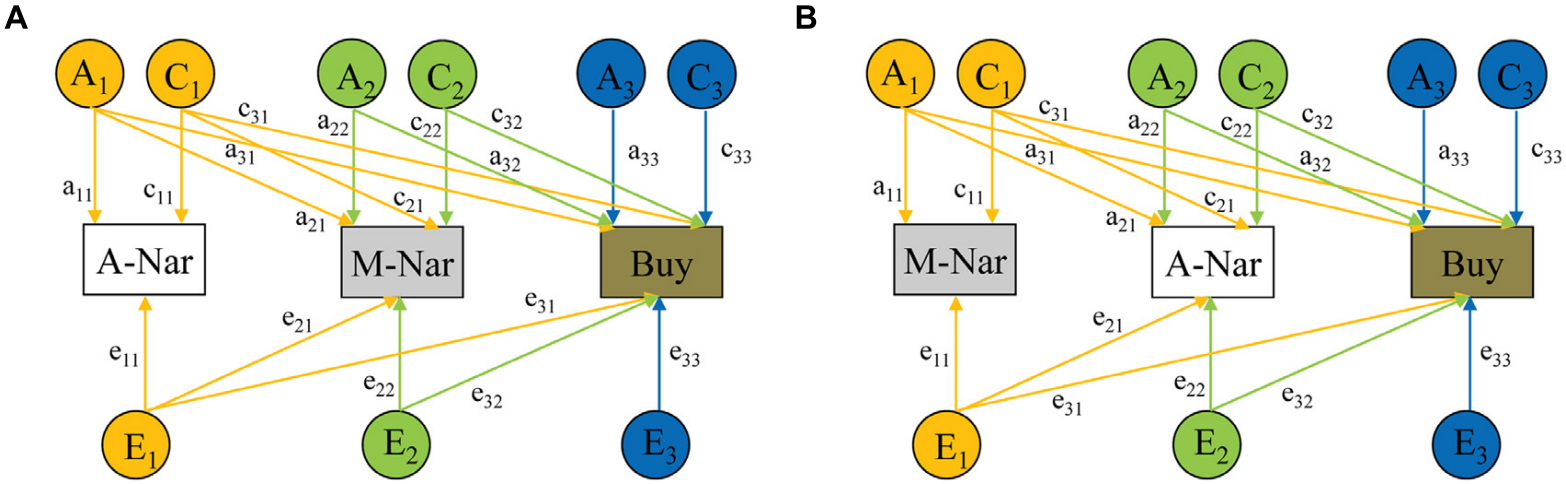
**Path diagram illustrating the multivariate model-fitting for maladaptive narcissism (M-Nar), adaptive narcissism (A-Nar), and impulsive buying (Buy). (A)** The model from adaptive narcissism to maladaptive narcissism and impulsive buying. **(B)** The model from maladaptive narcissism to adaptive narcissism and impulsive buying. Measured variables are depicted in rectangles. Latent factors A (additive genetic factor), C (shared environmental factor), and E (non-shared environmental factor) are presented in circles.

Finally, for the multivariate analysis of adaptive narcissism, maladaptive narcissism, and impulsive buying, we used Cholesky decomposition. The multivariate model parameterized the variances for and the covariances among adaptive narcissism, maladaptive narcissism, and impulsive buying into three groups of genetic and environmental effects (**Figure 2**). A_1_, C_1_, and E_1_ represent genetic, shared environmental, and non-shared environmental influences common to all measures; A_2_, C_2_, and E_2_ represent influences common to the second and the last variables in the model; and A_3_, C_3_, and E_3_ represent influences unique to the last variable. Notably, A_1_, C_1_, and E_1_ also include influences specific to the first variable in the model, whereas A_2_, C_2_, and E_2_ also include influences specific to the second variable in the model. We tested two Cholesky models. As personality theories typically hypothesize traits as causes of behaviors, rather than the reverse (Allport, 1937; McCrae and Costa, 1999), we entered impulsive buying last in both models. In one model (**Figure 2A**), we entered adaptive narcissism first and maladaptive narcissism thereafter, so that we could examine the specific genetic and environmental influences from maladaptive narcissism to impulsive buying. In the other model (**Figure 2B**), we entered maladaptive narcissism first and adaptive narcissism second, so that we could also examine the specific genetic and environmental influences from adaptive narcissism to impulsive buying. The two models operate in the same way except that the order of entering the variables is reversed. From the model paths, we can estimate genetic, shared, and non-shared environmental effects on adaptive narcissism, maladaptive narcissism, and impulsive buying. We also can estimate correlations between these effects (i.e., genetic, shared, and non-shared environmental correlations) based on the model paths. As with the previous analyses, the full ACE model and the sub-models were systematically tested.

We used the change in chi-square (χ^2^) and Akaike’s Information Criterion (AIC; Akaike, 1987) as model fit indices. A lower AIC value indicates better fit. To compare a sub-model with the full model, a significant chi-square difference suggests that the nested model performs worse than the full model, resulting in retaining the full model; otherwise, the nested model with fewer parameters fits better in terms of parsimony (Bollen, 1989; Kline, 1998). Hence, the better-fit model receives due consideration (Kline, 1998).

### Results

#### Descriptive Statistics

The overall NPI score (*M* = 13.64, *SD* = 6.05) was modestly related to impulsive buying (*r* = 0.23, *p* < 0.001). As in Study 1, maladaptive narcissism (*M* = 6.25, *SD* = 3.10) was significantly correlated with impulsive buying (*M* = 3.18, *SD* = 1.28; *r* = 0.29, *p* < 0.001), but adaptive narcissism (*M* = 4.90, *SD* = 2.52) was not (*r* = 0.09, *p* = 0.147). Consistent with Study 1, it was maladaptive but not adaptive narcissism that predicted impulsive buying, providing convergent evidence for our expectations. Inconsistent with Study 1, however, the relationship between overall narcissism and impulsive buying was significant.

#### Univariate Genetic Analysis

Because twins are perfectly correlated for age and for gender when of the same gender, variations associated with age or gender would inflate the correlation between twins. Following standard procedure, all measures were corrected for age and gender effects using multiple regression and standardized residuals were saved for genetic analyses (McGue and Bouchard, 1984).

##### Impulsive buying

On impulsive buying, the MZ twin correlation (0.49) was higher than the DZ correlation (0.33; **Table 1**), suggesting that it is heritable. Next, we examined heritability by fitting a series of univariate models (**Table 2**). The full ACE model showed a moderate heritability of 27% with shared (7%) and non-shared (67%) environments accounting for the rest of the individual difference. Removing either C (AE model, *p* = 0.708) or A (CE model, *p* = 0.227) did not significantly change the model fit. But the simultaneous exclusion of A and C (E model, *p* < 0.001) decreased the model fit. In addition, the AE model displayed the lowest AIC value within the full model (435.60) and all sub-models (AE: 433.74, CE: 435.06, E: 452.03) and was chosen for this reason (Bollen, 1989; Kline, 1998). According to the AE model, genetic and non-shared environmental factors accounted for 34 and 66% of the individual differences in impulsive buying, respectively. The results were comparable to previous findings (Bratko et al., 2013).

**Table 1:** Twin intraclass correlations (ICC).

Measure	ICC_MZ_	N_MZ_	ICC_DZ_	N_DZ_
Maladaptive narcissism	0.66 (0.53–0.75)	152	0.15 ($-$0.18–0.38)	151
Adaptive narcissism	0.55 (0.38–0.68)	152	0.31 (0.04–0.50)	149
Impulsive buying	0.49 (0.27–0.64)	127	0.33 (0.06–0.52)	142

*MZ, monozygotic twins; DZ, dizygotic twins. 95% confidence intervals are in parentheses.*

**Table 2:** Univariate genetic model-fitting.

					Change from full model					
Measure	Model	-2LL	df	AIC	Δχ^2^	Δdf	*p*	a^2^	c^2^	e^2^
Impulsive buying	ACE	1503.60	534	435.60				0.27 (0.00–0.47)	0.07 (0.00–0.35)	0.67 (0.53–0.83)
	AE	1503.74	535	433.74	0.14	1	0.708	0.34 (0.20–0.47)		0.66 (0.53–0.80)
	CE	1505.06	535	435.06	1.46	1	0.227		0.26 (0.15–0.37)	0.74 (0.63–0.85)
	E	1524.03	536	452.03	20.43	2	0.000			1.00 (1.00–1.00)
Maladaptive narcissism	ACE	1677.244	602	473.24				0.44 (0.25–0.55)	0.00 (0.00–0.14)	0.56 (0.45–0.69)
	AE	1677.244	603	471.24	0.00	1	1.000	0.44 (0.31–0.55)		0.56 (0.45–0.69)
	CE	1689.181	603	483.18	11.94	1	0.001		0.29 (0.19–0.39)	0.71 (0.61–0.81)
	E	1716.495	604	508.50	39.25	2	0.000			1.00 (1.00–1.00)
Adaptive narcissism	ACE	1651.486	599	453.49				0.37 (0.00–0.49)	0.00 (0.00–0.32)	0.63 (0.51–0.77)
	AE	1651.486	600	451.49	0.00	1	1.000	0.37 (0.24–0.49)		0.63 (0.51–0.76)
	CE	1654.94	600	454.94	3.45	1	0.063		0.28 (0.18–0.38)	0.72 (0.62–0.82)
	E	1680.057	601	478.06	28.57	2	0.000			1.00 (1.00–1.00)

*-2LL, twice the negative log-likelihood, the difference between -2LL of two models is subjected to chi-square (χ^2^) distribution. Two fit indices are reported: change in chi-square (Δχ^2^) and Akaike’s Information Criterion (AIC). Δ*df*, change in degrees of freedom (df). a^2^, c^2^, and e^2^ are proportion of variance due to additive genetic (A), shared environmental (C), and non-shared environmental effect (E). 95% confidence intervals are in parentheses. E, CE, and AE models are nested within ACE. The best-fit model is underlined.*

##### Overall narcissism

The MZ twin correlation was higher than that of DZ twins (MZ = 0.66, DZ = 0.36). The full ACE model found substantial genetic (47%) and non-shared environmental effects (53%), but no significant shared environmental effect (0%). Since the AE model fitted the data as well as the ACE model (*p* = 1.000), the AE model was preferable according to the parsimony principle (Bollen, 1989; Kline, 1998). Based on the AE model, overall narcissism was moderately heritable, with substantial influence drawn from non-shared environments^4^.

##### Maladaptive narcissism

For maladaptive narcissism, MZ twins (0.66) resembled each other two times more than DZ twins did (0.15; **Table 1**), suggesting significant genetic influence and trivial shared environmental influence. A series of univariate model-fitting confirmed this result (**Table 2**)^5^. The full ACE model identified a heritability of 44% and a non-shared environmental effect of 56%, with zero contribution from shared environment. The removal of the C component did not decrease the model fit (*p* = 1.000). But dropping A (CE model, *p* = 0.001) or both A and C (E model, *p* < 0.001) significantly reduced the model fit. Hence, the AE model was preferable (Bollen, 1989; Kline, 1998). In the AE model, the estimates for the genetic and non-shared environmental influences were the same as those in the ACE model.

##### Adaptive narcissism

On adaptive narcissism, MZ twins (0.55) resembled each other more than DZ twins did (0.31; **Table 1**), which suggested genetic influences. Subsequently, we employed univariate model-fitting to estimate the genetic and environmental contributions to the individual differences in adaptive narcissism (**Table 2**). Based on the results from the ACE model, 37% of individual differences in adaptive narcissism could be explained by genetic factors, with the other 63% attributed to non-shared environment and an estimate of zero for shared environment. The AE model fitted the data as well as the full ACE model (*p* = 1.000), whereas the CE (*p* = 0.063) and E (*p* < 0.001) models decreased the fitness at least to a marginal extent. Following the parsimony principle (Kline, 1998), the AE models provided the best account of the variances in adaptive narcissism.

#### Bivariate Genetic Analysis of Overall Narcissism and Impulsive Buying

We tested the full ACE model first and thereafter the AE, CE, and E models (**Table 3**). Compared with the full model, the AE model fitted the data equally well (*p* = 0.301). But the CE and E models (*p*s < 0.001) were significantly worse. In line with the univariate model-fitting, the AE model was optimal (**Figure 1B**). The AE model identified a medium genetic correlation (0.44, 95% CI: 0.19, 0.69), but a non-significant non-shared environmental correlation (0.02, 95% CI: -0.13, 0.17) between narcissism and impulsive buying. This outcome suggested that narcissism and impulsive buying share genetic sources to a considerable extent, whereas the non-shared environment underlying them is largely different.

**Table 3:** Bivariate model-fitting of impulsive buying and overall narcissism.

				Change from full model		
Model	-2LL	*df*	AIC	Δχ^2^	Δ*df*	*p*
ACE	3137.76	1132	873.76			
AE	3141.43	1135	871.43	3.66	3	0.301
CE	3150.89	1135	880.89	13.13	3	0.000
E	3210.02	1138	934.02	72.26	6	0.000

*-2LL, twice the negative log-likelihood; AIC, Akaike’s Information Criterion; Δχ^*2*^, change in chi-square; Δ*df*, change in degrees of freedom (df); A, additive genetic effects; C, shared environmental effects; E, non-shared environmental effects. E, CE, and AE models are nested within the ACE model. The best fitting model is underlined.*

#### Multivariate Genetic Analysis of Adaptive, Maladaptive Narcissism, and Impulsive Buying

**FIGURE 3:**
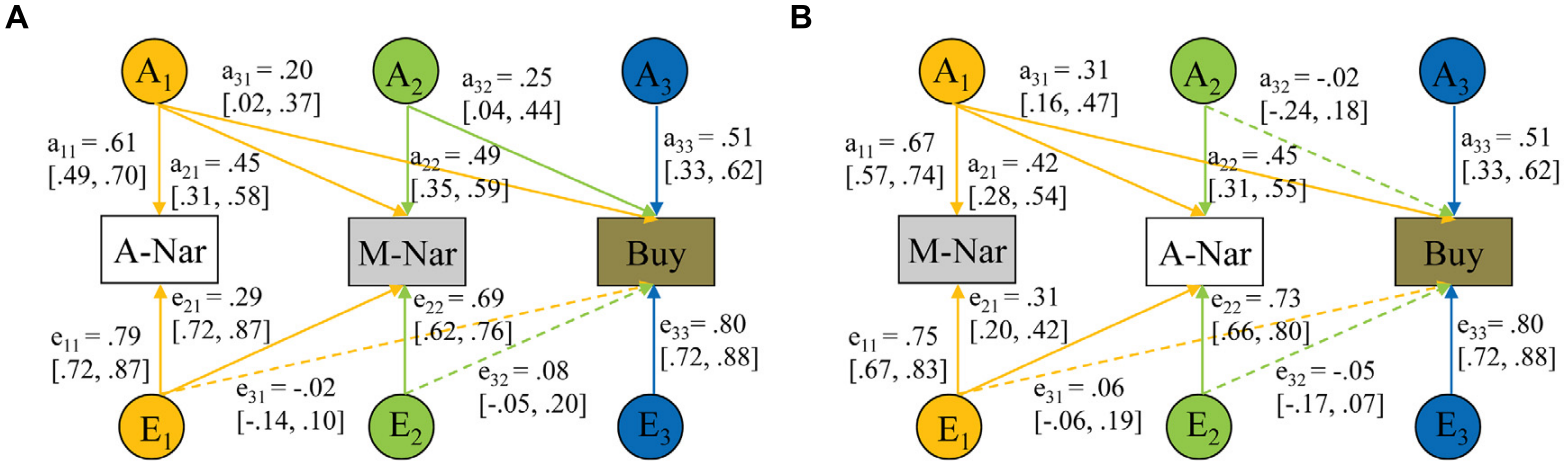
**The best-fitting multivariate model for maladaptive narcissism (M-Nar), adaptive narcissism (A-Nar), and impulsive buying (Buy). (A)** The model from adaptive narcissism to maladaptive narcissism and impulsive buying. **(B)** The model from maladaptive narcissism to adaptive narcissism and impulsive buying. Measured variables are depicted in rectangles. Latent factors A (additive genetic factor), C (shared environmental factor), and E (non-shared environmental factor) are presented in circles. All path estimates (95% confidence intervals) are standardized but unsquared. The non-significant path is represented by a dashed line.

Two multivariate models were applied to analyze the genetic and environmental influences from adaptive narcissism to maladaptive narcissism and impulsive buying (**Figure 2A**), and from maladaptive narcissism to adaptive narcissism and impulsive buying (**Figure 2B**), respectively. For each model, we tested the full ACE model first and thereafter the AE, CE, and E models (**Table 4**). Compared with the full model, the AE model fitted the data equally well (*p* = 0.861). But the CE and E models were significantly worse (*p*s < 0.01). In line with the univariate model-fitting, the AE model was optimal (**Figure 3**). It is worth noting that the two chosen AE models fitted the data equally well because they functioned in the same way on the same data, with the exception of the order of variables (i.e., adaptive and maladaptive narcissism).

**Table 4:** Multivariate model-fitting of impulsive buying, adaptive narcissism, and maladaptive narcissism.

				Change from full model		
Model	-2LL	*df*	AIC	Δχ^2^	Δ*df*	*p*
Adaptive narcissism → Maladaptive narcissism → Impulsive buying (**Figure 3A**)					
ACE	4629.18	1726	1177.18			
AE	4631.75	1732	1167.75	2.57	6	0.861
CE	4647.51	1732	1183.51	18.33	6	0.005
E	4707.73	1738	1231.73	78.55	12	0.000

Maladaptive narcissism → Adaptive narcissism → Impulsive buying (**Figure 3B**)					
ACE	4629.18	1726	1177.18			
AE	4631.75	1732	1167.75	2.57	6	0.861
CE	4647.51	1732	1183.51	18.33	6	0.005
E	4707.73	1738	1231.73	78.55	12	0.000

*-2LL, twice the negative log-likelihood; AIC, Akaike’s Information Criterion; Δχ^*2*^, change in chi-square; Δ*df*, change in degrees of freedom (df); A, additive genetic effects; C, shared environmental effects; E, non-shared environmental effects. E, CE, and AE models are nested within the ACE model. The best fitting model is underlined. Because the two sets of models functioned in the same way, except the order of variables (i.e., adaptive and maladaptive narcissism), on the same data, they provided the same fit to the data.*

As seen in **Figure 3**, the genetic effect on impulsive buying was composed of three parts. One part was caused by genetic influences common to all variables (A_1_), another part by genetic influences shared with maladaptive/adaptive narcissism (A_2_), and the remaining part by unique genetic influences (A_3_). Comparing **Figure 3A** and **Figure 3B**, we found that, (1) after controlling for adaptive narcissism (**Figure 3A**), genetic factors (i.e., A_2_) influencing maladaptive narcissism also exerted significant influence on impulsive buying (a_32_ = 0.25, 95% CI: 0.04, 0.44); (2) after controlling for maladaptive narcissism (**Figure 3B**), however, genetic factors (i.e., A_2_) influencing adaptive narcissism imparted trivial influence on impulsive buying (a_32_ = -0.02, 95% CI: -0.24, 0.18). Similarly, the non-shared environmental effect also included three parts. But only influence from the unique factor (E_3_) were significant (e_33_ = .80, 95% CI: 0.72, 0.88). Influences from the two shared factors (E_1_, E_2_) were limited (e_31_ and e_32_ ranged from -0.05 to 0.08, 95% CIs included zero).

In addition, between maladaptive narcissism and impulsive buying, the multivariate analysis identified a medium genetic correlation (*r*_g_ = 0.53, 95% CI: 0.27, 0.78), but a trivial non-shared environmental correlation (*r*_e_ = 0.08, 95% CI: -0.08, 0.23). This outcome suggested that maladaptive narcissism and impulsive buying share genetic sources to a considerable extent, whereas the non-shared environment underlying them is largely different. Regarding adaptive narcissism and impulsive buying, the analysis showed that there was a modest genetic correlation between them (*r*_g_ = 0.33, 95% CI: 0.04, 0.64). Since we identified no specific genetic influences from adaptive narcissism to impulsive buying after maladaptive narcissism was controlled, the genetic overlap between adaptive narcissism and impulsive buying was largely attributed to genetic influences shared with maladaptive narcissism (i.e., A_1_). We also found a minimal non-shared environmental correlation between adaptive narcissism and impulsive buying (-0.03, 95% CI: -0.17, 0.12). This finding indicated that non-shared environments underlying them are mostly different.

In summary, we found that the tendency for impulsive buying and overall narcissism as well as their association were heritable. As for the two components of narcissism, adaptive and maladaptive narcissism, they both were also heritable. Again, maladaptive narcissism but not adaptive narcissism predicted impulsive buying. Moreover, the association between maladaptive narcissism and impulsive buying had some genetic basis.

## Discussion

Impulsive buying is pervasive. This behavior can be attributed to situational triggers, personal dispositions, or both. In this research, we treated impulsive buying as a trait-like disposition and examined whether narcissism may predispose some individuals to be more likely to conduct impulsive buying. We found that the correlation between global narcissism and impulsive buying is significant, whereby individuals with high narcissism are more likely to engage in impulsive buying^6^. For two components of narcissism, we found that high maladaptive narcissism rather than high adaptive narcissism predicts high impulsive buying. Moreover, we learned that the relationship between global narcissism and impulsive buying as well as between maladaptive narcissism and impulsive buying have genetic bases. These results have important implications.

Impulsivity gives rise to a substantial portion of our daily purchases. Previous studies have revealed many predictors of impulsive buying, including age (Wood and Ahuvia, 1998), gender (Dittmar et al., 1996), culture (Kacen and Lee, 2002), affect (Verplanken et al., 2005), the Big Five Personality traits (Verplanken and Herabadi, 2001), self-control (Vohs and Faber, 2007), and others. We identified a novel predictor of impulsive buying, that is, narcissism, thus confirming our hypothesis. This finding matches previous findings that narcissists are impulsive (Vazire and Funder, 2006; but see Miller et al., 2009) and materialistic (Kasser and Ryan, 1996; Roberts and Robins, 2000) and that those individuals with a highly positive self-view are more likely to buy on impulse (Cash and Cash, 1982). As expected, maladaptive narcissism rather than adaptive narcissism is particularly predictive of impulsive buying. This result is in line with a large body of pre-existing findings whereby a negative outcome is usually associated with maladaptive narcissism rather than adaptive narcissism and thus adds to the mounting evidence that adaptive and maladaptive narcissism are distinct and have different functions (Emmons, 1984; Raskin and Terry, 1988; Watson and Biderman, 1993; Washburn et al., 2004; Barry et al., 2007; Ackerman et al., 2011; Hepper et al., 2014). In summary, our findings contribute novel detail to the portrait of an impulsive buyer.

More important, the discovery of the link between impulsive buying and narcissism is strengthened by the twin study. Past twin studies have demonstrated that both narcissism and the tendency for impulsive buying are heritable (Vernon et al., 2008; Bratko et al., 2013; Luo et al., 2014). We successfully replicated these findings in a twin sample from Beijing, China, with genetic factors accounting for 34% of the variations for impulsive buying, and 47, 37, and 44% of the variations for global narcissism, adaptive and maladaptive narcissism, respectively. We found significant genetic correlation between global narcissism and impulsive buying. As for narcissism’s two subcomponents, we did not find a significant relationship between adaptive narcissism and impulsive buying. We did discover, however, a significant relationship between maladaptive narcissism and impulsive buying, which is, moreover, genetically based. The genetic basis of a phenotypic correlation suggests the fundamental nature of the association, which may imply pleiotropy (i.e., one gene influences multiple traits), a circumstance in which genes that influence narcissism may also influence impulsive buying (Kovas and Plomin, 2006). This genetic correlation also suggests that genetic factors may affect narcissism and impulsive buying by influencing some common factors underlying them both. Existing evidence suggests this possibility. For instance, extraversion, neuroticism, and impulsivity are each found to be connected to both impulsive buying (Bratko et al., 2013) and the maladaptive components of narcissism (Corry et al., 2008; Hill and Roberts, 2012). Moreover, some of the connections have genetic bases (Vernon et al., 2008; Bratko et al., 2013). Hence, it is likely that genes influence some basic personality traits, further predisposing an individual to both narcissism and impulsive buying. Such hypotheses, however, still require additional genetic studies in the future.

Our results also showed that individual differences in narcissism and impulsive buying, together with their associations, are not entirely determined by genetic factors. Although shared environment contributes little, non-shared environment reveals substantial differences. This pattern is consistent with a large body of extant findings obtained from twin studies in personality and social psychology (Plomin et al., 2008). Individuals, including identical twins living in the same environment, differ in the ways they perceive and understand their environment, and therefore are influenced differently based on a particular environment (Hanscombe et al., 2010). Given the harmful consequences of impulsive buying, the pronounced impact of non-shared environment suggests the possibility of reducing the tendency for impulsive buying.

A series of theories and empirical findings have characterized narcissists as consumers who care more about the symbolic value than the utilitarian value of a product (Sedikides et al., 2007; Cisek et al., 2008; Lee et al., 2013). Our present studies have identified another feature of narcissistic consumers where they tend to buy on impulse. The unrestricted desire for material possessions as well as the preference for exclusive products may leave narcissists at financial risk in the long run. This possibility calls for the development of intervention strategies targeting narcissistic consumption. Since impulsive purchases contribute to almost half of daily human purchases (Nichols et al., 2001), being aware of a high impulsive buying tendency among high narcissists may be good for business promotion. Marketing practices may especially be designed to target these specific groups. To do so, of course, we need to differentiate adaptive narcissism from maladaptive narcissism.

Notably, the measures for adaptive and maladaptive narcissism only manifested moderate reliabilities. The low reliability, though consistent with previous findings (Barry et al., 2007; Barry and Malkin, 2010; Hepper et al., 2014), may reduce the power of detecting genetic influence because genetic analysis is based on correlations. Nevertheless, we still identified significant heritability for maladaptive narcissism as well as its association with impulsive buying, which suggests the potency of genetic influence on the one hand, and on the other hand, cautions that future replication is needed. As for the measure of impulsive buying, it manifested relatively high reliability, being comparable to the original study that constructed the scale (Rook and Fisher, 1995). Nevertheless, we should exercise caution recognizing that the scale might not reflect the complexity of impulsive buying. In addition, we also must be aware of the correlational nature of our present research. Although personality theories typically hypothesize traits as causes of behaviors, rather than the reverse (Allport, 1937; McCrae and Costa, 1999), future studies employing longitudinal design are needed to test possible causal relationships. Finally, as we have operationalized impulsive buying as the tendency to buy on impulse, one should keep in mind that a trait tendency may not necessarily result in real behavior (Rook and Fisher, 1995).

In summary, both phenotypic and genetic evidence suggested a connection between narcissism and impulsive buying. Being aware of the increasing tendency toward narcissism (of course including maladaptive narcissism) among young adults worldwide (Twenge et al., 2008; Cai et al., 2012), whether or not impulsive buying is on the rise constitutes an important topic for future research.

## Author Contributions

HC and XF conceived and designed the experiments. YS and YL collected the data. YL analyzed the data. HC and YL drafted the paper. YS and XF provided critical revisions. All authors approved the final version of the manuscript and agreed to account for all aspects of the work.

## Conflict of Interest Statement

The authors declare that the research was conducted in the absence of any commercial or financial relationships that could be construed as a potential conflict of interest.

## Footnotes

1 This news was retrieved from the official site of the Alibaba Group: http://alibabagroup.com/cn/news/article?news=p141112 [accessed December 01, 2014].

2 Throughout the article, “narcissism” refers to individual difference that is continuous within normal populations, i.e., subclinical narcissism.

3 For the convenience of terminology, we use “narcissist” to refer a normal person exhibiting relatively high subclinical narcissism.

4 These results were reported in Luo et al. (2014).

5 Although MZ correlations are more than twice as large as DZ correlations for maladaptive narcissism, we did not estimate the dominance genetic effect (ADE model) due to the lack of power arising from our relatively small sample (Martin et al., 1978). Another reason we did not test the ADE model is that previous twin studies of narcissism, all of which were measured with the NPI as we did, found that genetic effects on narcissism are additive rather than dominant (Vernon et al., 2008; Luo et al., 2014). In particular, Luo et al. (2014) and the present study are based on the same sample as well as the same measure of narcissism, i.e., NPI. As for the DE model, it was never tested given that a model with dominant genetic variance that lacks additive genetic variance is biologically implausible (Neale and Cardon, 1992).

6 The correlation between global narcissism and impulsive buying in Study 1 is positive but not significant. We conducted a meta-analysis to combine the correlation from Study 1 (*r* = 0.14, *N* = 112) and Study 2 (*r* = 0.23, *N* = 304). The weighted mean correlation across two studies is significant (*r* = 0.21, 95% CI: 0.12, 0.29).
